# What influences surgeons’ experience of surgical research?

**DOI:** 10.1186/1745-6215-14-S1-P80

**Published:** 2013-11-29

**Authors:** Cushla Cooper, David Beard, Andrew Carr

**Affiliations:** 1 University of Oxford, Oxford, UK

## 

Whilst there is evidence to suggest that surgeons recognise the need for research in surgery and a requirement to be familiar with research methods, including bias and design weaknesses, there remains a reluctance to participate in, and recruit to, surgical research. Compared to drug trials, surgical research offers some unique challenges and incorporation into standard surgical practice can be problematic. However, such characteristics remain largely unexplored. Retrospective exploration of surgeon experience with surgical research could help identify strengths and weaknesses of study protocols and contribute to the design of future surgical trials. The objective of this study was to explore the surgeons’ experiences with surgical research and use this information to develop a tool to be used to identify issues that could be addressed before surgeons participate in future surgical research.

Consultant Orthopaedic Surgeons who were involved in a large multicentre surgical trial were interviewed about their experiences with surgical research. Issues which arose from all interviews included their interpretation of equipoise, their treatment preferences and willingness to randomise, belief in the protocol design and study treatments, effects of NHS policies, patient care pathways and concerns about the direction of research. These findings have been incorporated into a new tool to be utilised prior to surgeon participation in a trial. The tool will be used to influence the study design, protocol content and and to pre-empt and highlight issues that may affect the integrity of a study.

